# Association between baseline neutrophil-to-lymphocyte ratio and short-term hearing recovery after glucocorticoid therapy in idiopathic sudden sensorineural hearing loss

**DOI:** 10.3389/fmed.2026.1792149

**Published:** 2026-04-02

**Authors:** Daxiong Ding, Jing Zhang, Shan Lin

**Affiliations:** 1 Department of Otolaryngology-Head and Neck Surgery, Affiliated Hospital of North Sichuan Medical College, Nanchong, Sichuan, China; 2 Department of Respiratory and Critical Care Medicine, Affiliated Hospital of North Sichuan Medical College, Nanchong, Sichuan, China

**Keywords:** glucocorticoid, hearing recovery, idiopathic sudden sensorineural hearing loss, neutrophil-to-lymphocyte ratio, prediction

## Abstract

**Objective:**

This study aimed to evaluate the predictive value of the neutrophil-to-lymphocyte ratio (NLR) for day-7 hearing recovery following oral glucocorticoid therapy in patients with idiopathic sudden sensorineural hearing loss (ISSNHL).

**Methods:**

We retrospectively reviewed the medical records of 214 patients with unilateral ISSNHL who were admitted to the Department of Otolaryngology–Head and Neck Surgery between 2022 and 2025. Multivariable logistic regression was used to assess the association between baseline NLR and day-7 recovery status (recovered vs. non-recovered), and E-values were calculated to evaluate the potential impact of unmeasured confounders. Stratified analyses and interaction tests were performed to examine potential effect modifications across predefined subgroups. Receiver operating characteristic (ROC) curve analysis was conducted to assess predictive performance, and the optimal NLR threshold was determined using the Youden index. To address potential overfitting and improve reproducibility, internal validation and calibration of the NLR-only model were performed using bootstrap resampling, including the optimism-corrected AUC, Brier score, calibration-in-the-large, and calibration slope.

**Results:**

A higher NLR was significantly associated with a lower likelihood of day-7 hearing recovery. In the fully adjusted model, the odds ratio (OR) for NLR was 0.40 [95% confidence interval (CI), 0.30–0.53]. The E-value was 2.54 (upper limit of the 95% CI, 2.09), supporting the robustness to unmeasured confounding factors. This association was generally consistent across subgroup analyses. ROC analysis showed strong predictive accuracy, with an apparent AUC of 0.90 (95% CI, 0.85–0.95). The optimal NLR cut-off was 4.59, with a specificity of 97.66% and a sensitivity of 80.23%. Bootstrap internal validation demonstrated minimal overfitting (optimism-corrected AUC 0.90) and satisfactory calibration (optimism-corrected calibration-in-the-large = 0.0039; calibration slope = 1.0032).

**Conclusion:**

Baseline NLR is a simple and readily available biomarker that predicts day-7 hearing recovery following oral glucocorticoid therapy in patients with ISSNHL. An elevated NLR is associated with a lower probability of recovery, supporting its potential utility for early risk stratification; nevertheless, external validation in independent cohorts is warranted.

## Introduction

Idiopathic sudden sensorineural hearing loss (ISSNHL), typically defined as hearing loss of at least 30 dB across three consecutive frequencies occurring within 72 h, is a common otologic emergency that primarily presents as acute sudden deafness (1). The incidence of ISSNHL is approximately 11–77 per 100,000 people per year (2). However, this statistic reflects only those who present for care in hospitals, and the true incidence is likely underestimated. In most cases, the etiology of ISSNHL remains idiopathic (3).

Recent advances in understanding the mechanisms underlying ISSNHL have suggested several potential etiological factors, including vascular lesions, autoimmunity, circulatory disorders, viral infections, and rupture of the labyrinthine membrane. Despite these insights, a comprehensive mechanism that fully explains ISSNHL pathogenesis remains elusive (3–5). Therefore, the pathogenesis of ISSNHL remains uncertain. In recent years, the pathogenesis of ISSNHL has been increasingly linked to the inflammatory response, which may lead to vascular endothelial damage and microvascular thrombosis, thereby increasing the risk of ischemia (6, 7). Since the cochlear blood supply is largely dependent on the single terminal labyrinthine artery, and cochlear hair cells have high oxygen demand and limited tolerance to hypoxia, microvascular thrombosis can disrupt inner ear circulation, ultimately leading to hearing loss (8, 9). Neutrophils and lymphocytes are key effector cells in the inflammatory process and have been implicated in ISSNHL. Masuda et al. found that neutrophil counts above normal reference values were associated with more severe hearing loss and poorer prognosis (6, 10). Therefore, inflammatory biomarkers may serve as valuable predictors of ISSNHL prognosis. One such biomarker, the neutrophil/lymphocyte ratio (NLR), has emerged as a sensitive indicator of systemic inflammation. An elevated NLR has been associated with poor outcomes in various conditions, including lung cancer, gastrointestinal tumors, respiratory disorders, and cardiovascular diseases (11–16).

While prior studies have explored the diagnostic and prognostic value of NLR in ISSNHL, differences in sample size, population characteristics, endpoint definitions, and follow-up duration have led to variable estimates of predictive performance, resulting in uncertainty among clinicians regarding how best to apply NLR in clinical practice (17–19). Additionally, evidence remains limited regarding whether baseline NLR can predict short-term hearing recovery after glucocorticoid therapy in patients with ISSNHL. Given that ISSNHL is an otolaryngological emergency in which early assessment, timely initiation of treatment, and risk stratification are critical for patient outcomes, there is an urgent need for simple and readily available biomarkers to help clinicians estimate the likelihood of post-treatment hearing recovery.

## Materials and methods

### Study population

The medical records of 214 patients with unilateral ISSNHL admitted to the Department of Otorhinolaryngology–Head and Neck Surgery at the Affiliated Hospital of North Sichuan Medical College between January 2022 and June 2025 were retrospectively analyzed. This retrospective study was approved by the Ethics Committee of the Affiliated Hospital of North Sichuan Medical College (Approval No. 2025ER285-1).

### Eligibility criteria

ISSNHL was defined in accordance with the 2019 American Academy of Otolaryngology–Head and Neck Surgery Clinical Practice Guideline as a sudden sensorineural hearing loss of ≥ 30 dB affecting at least three consecutive frequencies within 72 h, without an identifiable etiology after routine clinical assessment (1).

The exclusion criteria were: (1) systemic conditions that could influence inflammatory markers or hearing prognosis, including infectious diseases, autoimmune diseases, hematologic disorders, chronic liver disease, renal disease, tumors, and other major systemic diseases; (2) conditions or histories suggestive of non-idiopathic hearing loss, including neurological dysfunction, craniocerebral trauma, prior otologic surgery, drug-induced ototoxicity, noise-induced hearing loss, or Ménière’s disease; and (3) prior use of systemic hormones, antibiotics, or immunosuppressive drugs outside the hospital before admission.

### Treatment protocol

All patients received systemic glucocorticoid therapy and routine symptomatic supportive care during hospitalization. Specifically, oral prednisone was administered at a dosage of 1 mg/kg/day for 7 days. Adjunctive therapies were prescribed according to routine clinical practice to improve microcirculation (G. biloba extract) and provide neurotrophic support (adenosylcobalamin) (1, 20).

### Blood sampling and laboratory measurements

Peripheral venous blood samples were collected at admission and before the initiation of glucocorticoid therapy, as part of routine clinical care. Laboratory variables extracted from medical records included neutrophil count, lymphocyte count, blood glucose, total cholesterol, triglycerides, fibrinogen, and homocysteine.

The NLR was calculated as the absolute neutrophil count divided by the absolute lymphocyte count from the complete blood count at admission. All laboratory results used in the analysis were obtained before treatment initiation to minimize potential treatment-related changes in the inflammatory indices.

### Audiological assessment

Baseline audiological evaluations at admission were extracted from the audiology reports. Pure-tone audiometry was performed according to the routine departmental practice by certified audiologists using calibrated equipment. Hearing thresholds were obtained for the affected ear and used to determine the severity of hearing loss. The categorical variable “hearing loss ≥ 60 dB HL” was defined based on the baseline audiometric assessment of the affected ear recorded in the audiology report. Tinnitus status at presentation was obtained from the medical record and/or audiology documentation.

To evaluate treatment response, patients underwent repeat audiological assessments 7 days after oral glucocorticoid therapy, and hearing changes were determined by comparing follow-up results with baseline findings. Treatment efficacy was categorized as follows: (1) cured, hearing at the affected frequencies returned to normal or to the level of the contralateral ear or to the pre-disease level; (2) obvious effect, mean improvement in hearing thresholds across the affected frequencies > 30 dB; (3) effective, mean improvement of 15–30 dB; and (4) ineffective, mean improvement of < 15 dB (20). Patients were further divided into recovered (cured/obvious effect/effective) and non-recovered (ineffective) groups for subsequent analyses.

### Statistical analysis

Continuous variables are expressed as mean ± standard deviation, while categorical variables are presented as numbers (percentages). Comparisons between groups for continuous variables were performed using the *t*-test or Wilcoxon rank-sum test, while categorical variables were analyzed using the chi-squared test or Fisher’s exact test. The primary endpoint was day-7 recovery status (recovered vs. non-recovered) after the initiation of oral glucocorticoid therapy. Logistic regression was employed to evaluate the association between baseline NLR and day-7 recovery status. Covariates were primarily pre-specified *a priori* based on clinical relevance and prior literature, and variables were additionally retained if they materially altered the estimated NLR–outcome association (≥ 10% change in the odds ratio) (21, 22). Three regression models were used: Model I was adjusted for sex and age; Model II was adjusted for sex, age, diabetes, hypertension, hearing loss ≥ 60 dB HL, tinnitus, blood glucose, total cholesterol, triglycerides, homocysteine, and fibrinogen; and Model III was fitted using penalized logistic regression to reduce potential overfitting and improve generalizability. For Model III, the ridge (L2) and LASSO (L1) penalized logistic regression models were applied with the same candidate predictors as in Model II, and the penalty strength was selected using 10-fold cross-validation. The 10-fold cross-validated out-of-fold AUC was reported as an estimate of internal generalization performance. Bootstrap internal validation was performed (*B* = 1,000) to estimate optimism and obtain optimism-corrected performance measures, including AUC and Brier score. Model calibration was assessed using calibration-in-the-large (CITL; calibration intercept) and calibration slope, and a bootstrap-corrected calibration curve was generated.

The potential impact of unmeasured confounding factors on the observed association was assessed by calculating E-values (23, 24). Stratified analyses and interaction tests were conducted to evaluate the effect modification by prespecified subgroup variables. Discrimination was assessed using the area under the receiver operating characteristic curve (AUC), and the optimal threshold was determined using the Youden index. Internal validation was performed using bootstrap resampling (*B* = 1,000) to estimate optimism and obtain optimism-corrected performance measures. Overall accuracy was assessed using the Brier score. Calibration was evaluated using calibration-in-the-large (calibration intercept) and calibration slope, and a bootstrap-corrected calibration curve was generated. To explore potential non-linear associations, NLR was modeled using restricted cubic splines with four knots placed at the 5th, 35th, 65th, and 95th percentiles. The spline model was fitted within a multivariable logistic regression adjusted for age, sex, diabetes, hypertension, tinnitus, hearing loss ≥ 60 dB HL, fibrinogen, glucose, total cholesterol, triglycerides, and homocysteine. Non-linearity was assessed using a likelihood ratio test comparing the spline model with a model that included the NLR as a linear term. All analyses were conducted using R (version 4.2.2), with a two-sided *P* < 0.05 was considered statistically significant.

## Results

### Characteristics of the study population

A total of 214 patients with ISSNHL were included in the study and divided into recovered and non-recovered groups based on their response to treatment. The patients in the non-recovered group were older than those in the recovered group (mean age 51.95 vs. 45.34 years, *P* = 0.003), with no significant differences between the groups in sex or hypertension. The NLR was significantly lower in the recovered group than in the non-recovered group (mean NLR 4.08 vs. 7.49, *P* < 0.001) (Table 1). Among the remaining laboratory indices, neutrophil counts and levels of blood glucose, total cholesterol, triglycerides, homocysteine, and fibrinogen were significantly lower in the recovered group than in the non-recovered group. In contrast, the lymphocyte count was significantly higher in the recovered group (*P* < 0.001). Additionally, hearing loss ≥ 60 dB HL and tinnitus were significantly more common in the non-recovered group than in the recovered group (*P* < 0.001).

**TABLE 1:** Characteristics of participants.

Variables	All (*N* = 214)	Non-recovered group (*N* = 128)	Recovered group (*N* = 86)	*P*-value
Age (years)	49.29 ± 16.24	51.95 ± 13.00	45.34 ± 19.54	0.003
Sex				0.145
Male	142 (66.36%)	80 (62.50%)	62 (72.09%)	
Female	72 (33.64%)	48 (37.50%)	24 (27.91%)	
NLR	6.12 ± 2.76	7.49 ± 2.19	4.08 ± 2.20	< 0.001
Neutrophil (10^9^/L)	7.11 ± 2.29	7.94 ± 2.09	5.87 ± 2.00	< 0.001
Lymphocyte (10^9^/L)	1.35 ± 0.54	1.16 ± 0.43	1.64 ± 0.57	< 0.001
Glucose (mmol/L)	5.50 ± 1.47	5.69 ± 1.53	5.22 ± 1.32	0.021
Total cholesterol (mmol/L)	4.90 ± 1.07	5.13 ± 0.98	4.55 ± 1.10	< 0.001
Triglyceride (mmol/L)	1.11 ± 0.70	1.29 ± 0.77	0.83 ± 0.44	< 0.001
Homocysteine (μmol/L)	13.06 ± 3.91	13.90 ± 4.41	11.82 ± 2.61	< 0.001
Fibrinogen (g/L)	2.93 ± 0.76	3.07 ± 0.85	2.72 ± 0.53	< 0.001
Hearing loss ≥ 60 dB				< 0.001
No	94 (43.93%)	28 (21.88%)	66 (76.74%)	
Yes	120 (56.07%)	100 (78.12%)	20 (23.26%)	
Tinnitus				< 0.001
No	105 (49.07%)	33 (25.78%)	72 (83.72%)	
Yes	109 (50.93%)	95 (74.22%)	14 (16.28%)	
Hypertension				0.138
No	134 (62.62%)	75 (58.59%)	59 (68.60%)	
Yes	80 (37.38%)	53 (41.41%)	27 (31.40%)	
Type 2 diabetes				< 0.001
No	136 (63.55%)	68 (53.12%)	68 (79.07%)	
Yes	78 (36.45%)	60 (46.88%)	18 (20.93%)	

NLR, neutrophil-to-lymphocyte ratio.

### The association between baseline NLR and day-7 hearing recovery after glucocorticoid therapy

Logistic regression analyses showed that a higher baseline NLR was significantly associated with a lower likelihood of day-7 hearing recovery following oral glucocorticoid therapy. In the fully adjusted model (Model II), the odds ratio (OR) for NLR was 0.40 (95% confidence interval [CI], 0.30–0.53; *P* < 0.001) (Table 2), indicating that each 1-unit increase in NLR was associated with approximately a 60% decrease in the odds of recovery. Consistent findings were observed in penalized regression models: the ridge model yielded an OR of 0.53 (95% CI, 0.44–0.61; *P* < 0.001) and the LASSO model yielded an OR of 0.49 (95% CI, 0.35–0.60; *P* < 0.001) (Table 2), supporting the robustness of this inverse association between NLR and recovery.

**TABLE 2:** Association between baseline NLR and day-7 recovery response to glucocorticoid therapy.

Outcome: effective	OR	95% CI	*P*-value
Crude	0.46	0.37–0.57	< 0.001
Model I	0.42	0.33–0.54	< 0.001
Model II	0.40	0.30–0.53	< 0.001
Model III			
Ridge	0.53	0.44–0.61	< 0.001
LASSO	0.49	0.35–0.60	< 0.001

Model I was adjusted by age, sex. Model II was adjusted by age, sex, type 2 diabetes, hypertension, tinnitus, hearing loss ≥ 60 dB, fibrinogen, glucose, total cholesterol, triglyceride, homocysteine. Ridge and LASSO models included the same candidate predictors as Model II; the penalty parameter was selected by 10-fold cross-validation. For the ridge and LASSO models, odds ratios for NLR are reported per 1-unit increase in NLR on the original scale, and 95% confidence intervals were obtained using bootstrap resampling (*B* = 1000). CI, confidence interval; NLR, neutrophil-to-lymphocyte ratio; OR, odds ratio.

The potential impact of unmeasured confounding factors on the observed association was assessed using *E*-values (*E*-value 2.54; upper limit of the 95% CI, 2.09), suggesting moderate robustness to unmeasured confounding. Subgroup analyses and interaction tests indicated that the association between NLR and recovery was generally consistent across the predefined subgroups (Table 3). Although interactions were observed for hypertension, diabetes mellitus, hearing loss ≥ 60 dB HL, triglycerides, and blood glucose, these results should be interpreted cautiously because some subgroup sample sizes were limited, which reduced statistical precision and limited direct comparisons.

**TABLE 3:** Subgroup analyses of the association between baseline NLR and day-7 hearing recovery after glucocorticoid therapy.

X = NLR	N	Effective (OR, 95% CI)	*P*-value	Interaction *P*-value
Age (years)				0.2663
< 65	175	0.54 (0.40–0.72)	<0.0001	
≥ 65	39	0.00 (0.00–Inf)	0.9999	
Sex				0.1456
Male	142	0.00 (0.00–1.57)	0.0600	
Female	72	0.57 (0.36–0.89)	0.0126	
Hypertension				0.0235
No	134	0.51 (0.34–0.76)	0.0009	
Yes	80	0.00 (0.00–Inf)	0.999	
Type 2 diabetes				0.0058
No	136	0.50 (0.31–0.79)	0.0032	
Yes	78	0.00 (0.00–Inf)	0.9997	
Hearing loss ≥ 60 dB				0.0002
No	94	0.00 (0.00–Inf)	0.9997	
Yes	120	0.10 (0.01–1.27)	0.0761	
Tinnitus				0.6616
No	105	0.37 (0.18–0.76)	0.0067	
Yes	109	0.00 (0.00–6.80)	0.1334	
Total cholesterol				0.3524
< 5.72	169	0.37 (0.22–0.62)	0.0002	
≥ 5.72	45	0.02 (0.00–Inf)	1.0000	
Triglyceride				0.0038
< 1.8	179	0.49 (0.35–0.69)	<0.0001	
≥ 1.8	35	0.00 (0.00–Inf)	1.0000	
Homocysteine				–
< 25	214	0.42 (0.28–0.62)	<0.0001	
≥25	0	–	–	
Fibrinogen				0.9997
< 4	193	0.44 (0.30–0.65)	<0.0001	
≥ 4	21	1.00 (0.00–Inf)	1.0000	
Glucose				0.0025
< 6.11	155	0.19 (0.11–0.33)	<0.0001	
≥ 6.11	59	0.68 (0.47–0.99)	0.0449	

Adjusted by age, sex, type 2 diabetes, hypertension, tinnitus, hearing loss ≥ 60 dB, fibrinogen, glucose, total cholesterol, triglyceride, homocysteine except for the subgroup variable. CI, confidence interval; NLR, neutrophil-to-lymphocyte ratio; OR, odds ratio.

### Predictive value of NLR for day-7 hearing recovery after glucocorticoid therapy

**FIGURE 1:**
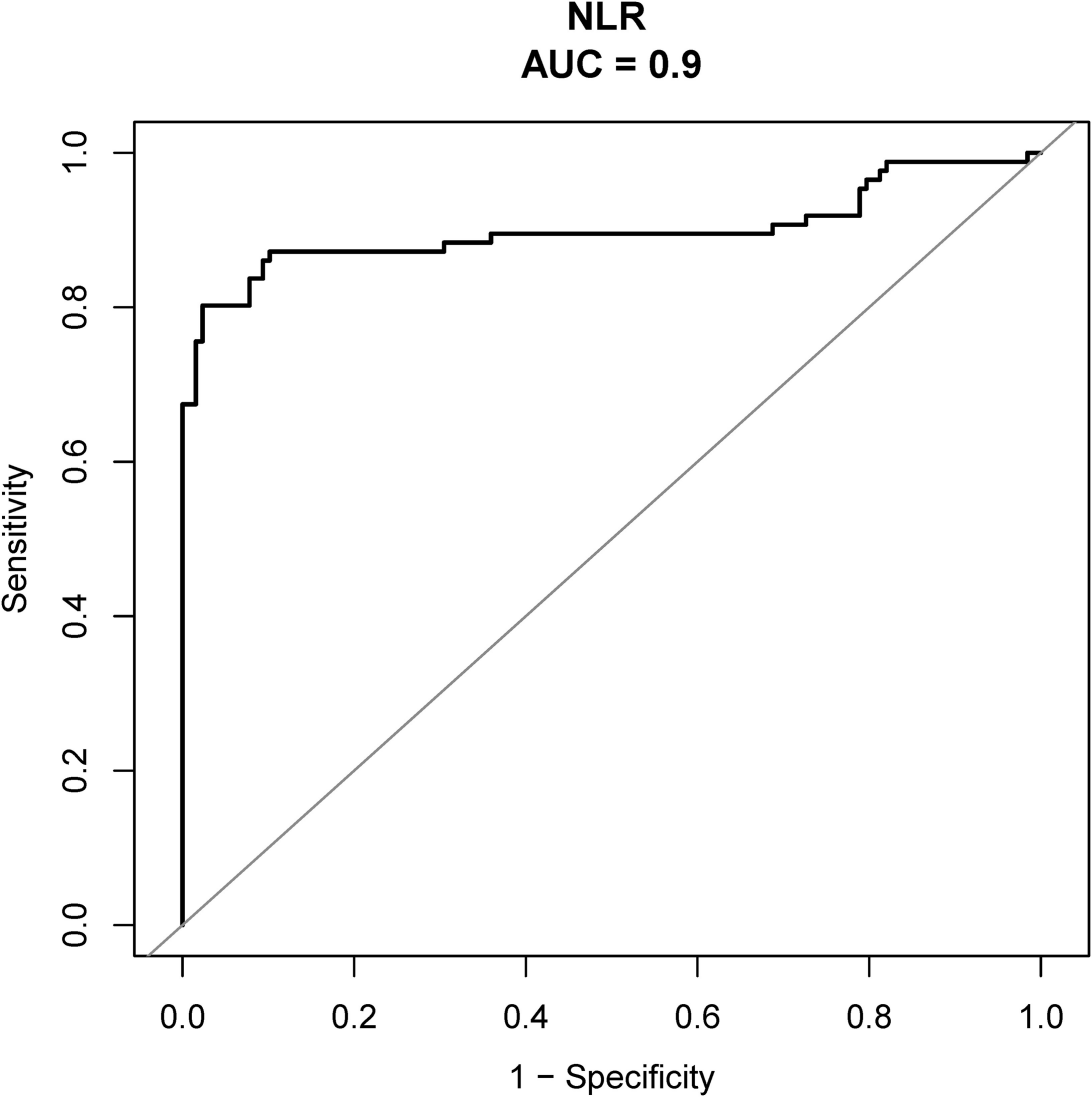
Receiver operating characteristic curve of baseline NLR for predicting day-7 hearing recovery after glucocorticoid therapy. NLR, neutrophil-to-lymphocyte ratio.

ROC curve analysis was performed to evaluate the ability of baseline NLR to predict day-7 hearing recovery after oral glucocorticoid therapy. NLR showed strong discriminative ability, with an apparent AUC of 0.90 (95% CI, 0.85–0.95) (Table 4 and Figure 1). Using the Youden index, the optimal cutoff for NLR was 4.59, yielding a specificity of 97.66%, a sensitivity of 80.23%, a positive likelihood ratio of 34.23, and a negative likelihood ratio of 0.20 (Table 4).

**TABLE 4:** Predictive performance of baseline NLR for day-7 hearing recovery after glucocorticoid therapy.

Variable	Cut-off value	AUC (95% CI)	Threshold value	Specificity	Sensitivity	Positive likelihood ratio	Negative likelihood ratio
NLR	4.59	0.90 (0.85–0.95)	4.59	0.98	0.80	34.23	0.20

AUC, area under the curve; CI, confidence interval; NLR, neutrophil-to-lymphocyte ratio.

To address concerns regarding potential overfitting, internal validation was conducted using bootstrap resampling (*B* = 1000). The optimism-corrected AUC was 0.9008, indicating negligible optimism. Model accuracy was supported by a low Brier score (apparent, 0.1269; optimism-corrected, 0.1288). Calibration was satisfactory, with calibration-in-the-large close to zero (optimism-corrected CITL = 0.0039) and a calibration slope close to one (optimism-corrected slope = 1.0032), suggesting good agreement between the predicted and observed probabilities. Bootstrap-corrected calibration curves are shown in Supplementary Table S1 and Supplementary Figure S1.

### Non-linear association between NLR and day-7 recovery (restricted cubic spline analysis)

**FIGURE 2:**
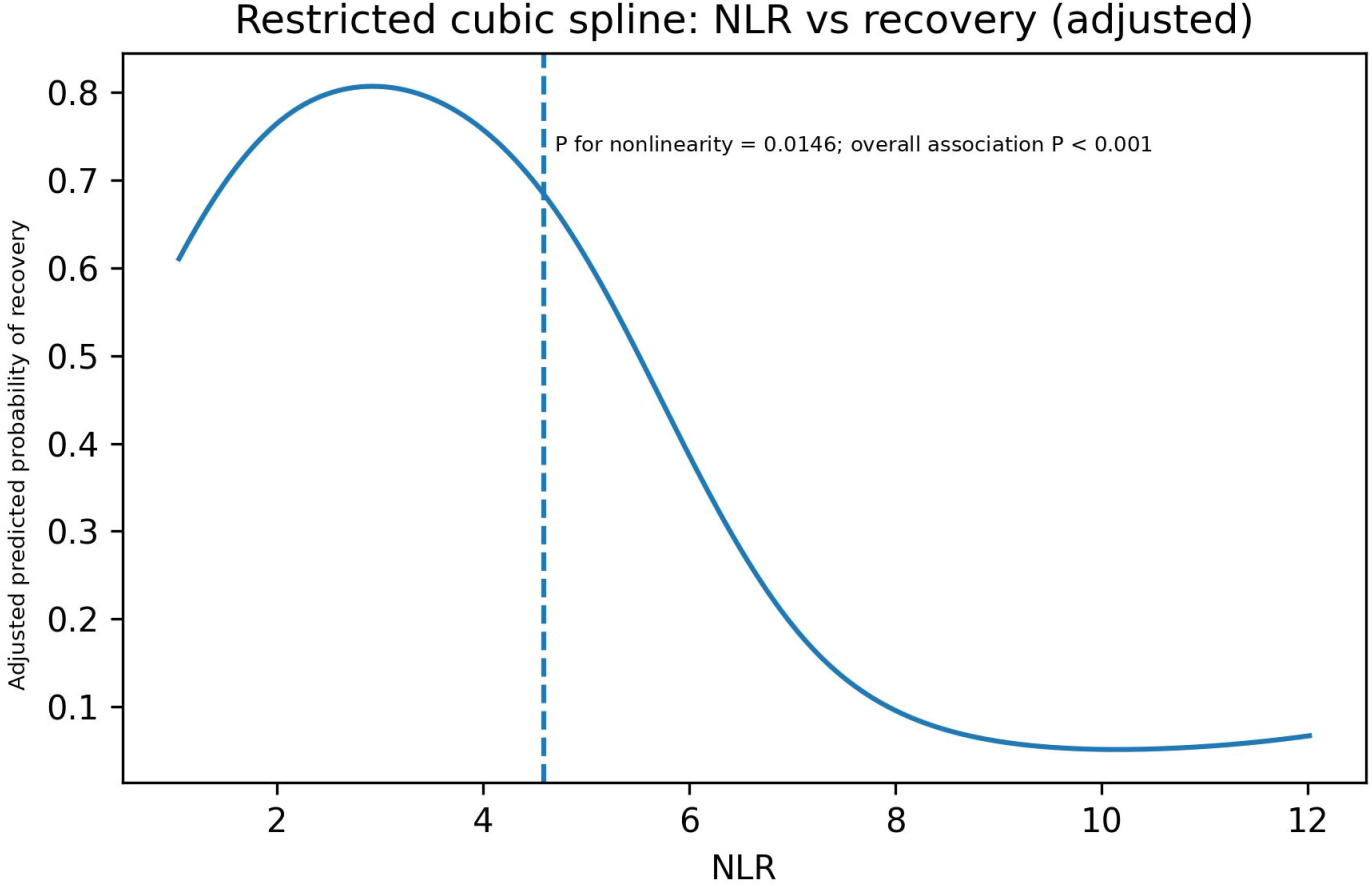
Restricted cubic spline analysis of the non-linear association between baseline NLR and day-7 hearing recovery after glucocorticoid therapy. NLR, neutrophil-to-lymphocyte ratio.

Restricted cubic spline analyses demonstrated a significant non-linear association between baseline NLR and day-7 recovery after adjustment for the Model II covariates (*P* for non-linearity = 0.0146; overall association *P* < 0.001) (Figure 2). The adjusted recovery probability peaked at approximately NLR ≈ 2.9 and declined progressively with higher NLR, with a steeper decrease when NLR exceeded approximately 5.

## Discussion

Idiopathic sudden sensorineural hearing loss is a common otologic emergency. Although the exact pathogenesis remains unclear, microcirculatory disturbances, prothrombotic susceptibility, and chronic inflammation are commonly proposed as potential causes. Timely recognition and prompt treatment are crucial, as medical interventions can often restore hearing and reduce tinnitus, thereby significantly improving patients’ quality of life. Therefore, simple and easy-to-use predictors of treatment outcomes are valuable for guiding clinical decisions. In the present study, baseline neutrophil-to-lymphocyte ratio (NLR) was inversely associated with day-7 hearing recovery after guideline-based oral glucocorticoid therapy. Patients with higher NLR were less likely to recover, and an NLR threshold of 4.59 showed strong discriminatory ability for predicting non-recovery. These findings suggest that clinicians should consider closer monitoring and early risk stratification for patients with an elevated NLR, although treatment decisions should be individualized and the findings require confirmation in future prospective studies.

Recent reviews have highlighted vascular mechanisms, such as cochlear ischemia and microvascular compromise, as plausible contributors to ISSNHL and have emphasized that inflammatory and thrombotic susceptibility may intersect with these pathways (25). In line with this framework, inflammation-related microvascular injury may contribute to ISSNHL via endothelial dysfunction, microthrombosis, and ischemic changes within the cochlear microcirculation (26). As a composite marker of systemic inflammation, the NLR is attractive because it is inexpensive, widely available, and potentially more stable than single leukocyte components. Prior studies have demonstrated the prognostic utility of NLR in a range of diseases (11–16). Mechanistically, neutrophil predominance may reflect heightened innate immune activation and endothelial injury, whereas relative lymphopenia may indicate impaired adaptive immune regulation, thereby favoring a pro-inflammatory milieu that could exacerbate microvascular compromise in the inner ear. Consistent with this rationale, previous studies have reported that SSNHL prognosis is negatively correlated with the neutrophil count and positively correlated with the lymphocyte count (27, 28). In addition, a recent systematic review of adult SSNHL biomarkers concluded that inflammatory indices derived from routine blood tests, particularly the NLR, platelet-to-lymphocyte ratio, and fibrinogen level, are among the most consistently informative and accessible markers for diagnostic and prognostic assessment (29).

Our findings are consistent with earlier clinical studies showing that NLR is elevated in patients with ISSNHL and lower in those who recover than in those who do not (30–33). Wu et al. also reported a strong discriminatory performance for NLR (AUC close to 0.90) with a defined threshold, further supporting its potential value in risk stratification (34). Similarly, meta-analyses have suggested that an elevated NLR is associated with poorer recovery in ISSNHL and that this association persists across different treatment regimens and follow-up durations, although causal inference remains limited (19, 35). More recently, a meta-analysis of 26 studies further supported the association between higher NLR and non-recovery and suggested that NLR may serve as a cost-effective prognostic marker, although heterogeneity across studies remains (36). Beyond inflammatory markers alone, emerging studies also suggest that metabolic profiles and systemic inflammatory status are jointly related to hearing recovery, supporting the clinical rationale for adjusting for comorbidities and biochemical variables in multivariable models (37).

To enhance clinical interpretability and address concerns about relying on a single cutoff, we further examined the dose–response pattern between NLR and recovery. Restricted cubic spline analyses demonstrated a significant non-linear relationship between NLR and day-7 recovery (*P* for non-linearity = 0.0146; overall association *P* < 0.001), indicating that the probability of recovery varies across the NLR range rather than changing uniformly. In addition, because the apparent AUC, specificity, and sensitivity were relatively high compared with those reported in some previous studies, we performed bootstrap internal validation and calibration analyses for the NLR-only model. These analyses showed minimal optimism with an optimism-corrected AUC close to 0.90 and satisfactory calibration (calibration-in-the-large near 0 and a calibration slope near 1), supporting the robustness of the model performance within our dataset. Nonetheless, because the cutoff was derived within the same cohort, the classification metrics may still be optimistic and should be interpreted as internally validated estimates rather than as definitive clinical thresholds. Notably, non-linear associations between NLR and recovery have also been reported in recent cohorts using smooth-curve approaches, supporting the premise that modeling NLR flexibly may better capture its clinical signal (38).

From a practical standpoint, NLR is available at presentation from a routine complete blood count and is best viewed as a risk-stratification and care-pathway tool, rather than a standalone trigger for changing therapy. In clinical practice, an elevated baseline NLR can be incorporated into the initial assessment to support structured follow-up planning and shared decision-making. Patients with higher NLR can be counseled as being at increased risk of short-term non-recovery, with emphasis on adherence, early reassessment, and timely escalation when recovery is incomplete. Given the high specificity observed at the ROC-derived threshold in our cohort, a markedly elevated NLR may serve as a “rule-in” signal to prioritize early repeat audiometry and to minimize delays in management steps that depend on documenting incomplete recovery. In particular, guideline-based care emphasizes reassessment and consideration of additional interventions when recovery is insufficient; therefore, using NLR to flag higher-risk patients may help ensure that they remain within appropriate time windows for next-step management and are not lost to follow-up. Because recovery is multifactorial, NLR should be interpreted alongside baseline hearing severity, comorbidities, and symptom profile. Future work should focus on translating NLR into actionable risk bands (e.g., low/intermediate/high risk informed by the observed non-linear dose–response), rather than relying solely on a single cut-off. Beyond prospective validation, several steps could improve clinical utility: developing and externally validating a parsimonious risk tool that combines NLR with a small set of readily available clinical variables and evaluating its clinical net benefit using decision-curve analysis; testing decision impact and implementation strategies (e.g., electronic health record prompts or standardized follow-up bundles for high-risk patients) to determine whether NLR-informed workflows improve reassessment timeliness and patient-centered outcomes; assessing whether serial NLR measurements add incremental prognostic value; and using NLR-defined strata to enrich or stratify pragmatic trials that evaluate intensified monitoring or adjunctive strategies in patients at higher risk of non-recovery.

This study has several limitations. First, its retrospective, single-center design may have been subject to selection bias and residual confounding factors. Although we adjusted for multiple clinically relevant covariates, performed sensitivity analyses, and quantified potential unmeasured confounding using *E*-values, causal inference could not be established. Second, although all patients received a guideline-based prednisone regimen (1 mg/kg/day for 7 days), the overall treatment protocol may not have been fully standardized in real-world practice. Variations in supportive therapies (e.g., microcirculation-improving or neurotrophic medications), timing of treatment initiation, and other unmeasured clinical management factors could have influenced the recovery outcomes. Third, several potentially important variables (e.g., body mass index, smoking status, and detailed medication history) were not available, which may have influenced the observed associations. Fourth, the outcomes were assessed on day 7, and long-term hearing recovery could not be evaluated. Finally, the generalizability and clinical applicability of the proposed NLR threshold and performance metrics remain unclear. Because the cutoff was derived using the Youden index within the same cohort and the study was conducted in a single-center setting with a short-term endpoint, the observed AUC and classification indices may be partly optimistic and influenced by spectrum effects. Although bootstrap internal validation and calibration analyses suggested minimal optimism and good calibration, external validation in independent, multicenter cohorts is required before clinical implementation.

## Conclusion

In summary, baseline NLR is a simple and accessible biomarker associated with day-7 hearing recovery following glucocorticoid therapy in patients with ISSNHL. The observed non-linear dose–response pattern and internally validated performance support its potential role in early risk stratification. Prospective multicenter studies with standardized treatment pathways and longer follow-up periods are warranted to confirm the generalizability and clinical utility of our findings.

## Data Availability

The original contributions presented in this study are included in this article/Supplementary material, further inquiries can be directed to the corresponding author.

## Supplementary material

Supplementary Figure S1 Bootstrap-corrected calibration curve for the NLR-only model predicting day-7 hearing recovery. The model was fitted using logistic regression with baseline NLR as a continuous predictor. Calibration was assessed using non-parametric bootstrap resampling (*B* = 1,000). The plot shows the relationship between predicted probabilities and observed outcomes, with the 45° line indicating perfect calibration.
