# Supplementary figures and images for: Association between baseline neutrophil-to-lymphocyte ratio and short-term hearing recovery after glucocorticoid therapy in idiopathic sudden sensorineural hearing loss
Source: Front Med (Lausanne). 2026 Apr 2;13:1792149. doi: 10.3389/fmed.2026.1792149 (PMC13083196; doi:10.3389/fmed.2026.1792149)

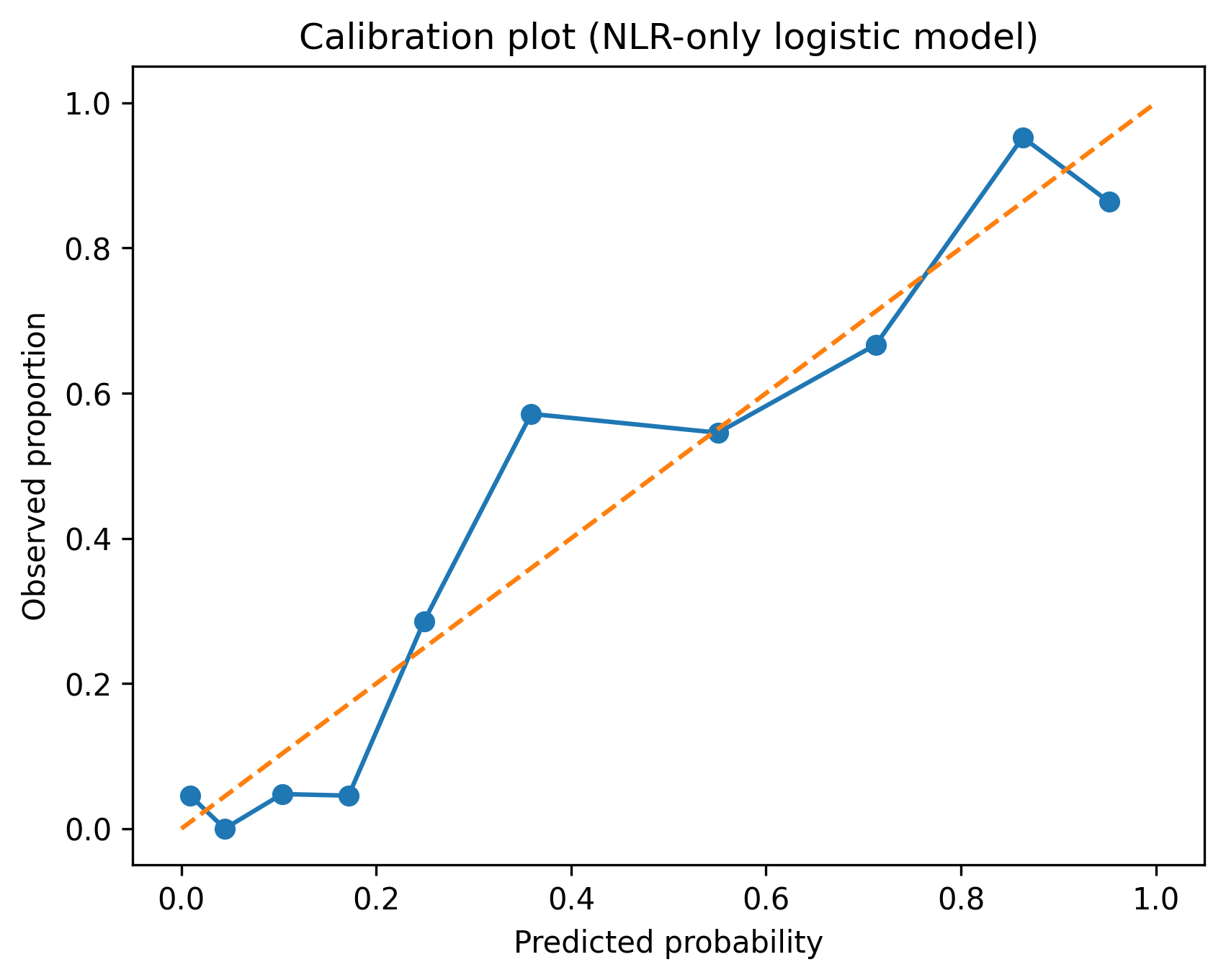

Supplement: Supplementary Figure S1 — Bootstrap-corrected calibration curve for the NLR-only model predicting day-7 hearing recovery. The model was fitted using logistic regression with baseline NLR as a continuous predictor. Calibration was assessed using non-parametric bootstrap resampling (B = 1,000). The plot shows the relationship between predicted probabilities and observed outcomes, with the 45° line indicating perfect calibration. [file Image_1.png]
